# Supplementary material for: Mash Screen: high-throughput sequence containment estimation for genome discovery
Source: Genome Biol. 2019 Nov 5;20:232. doi: 10.1186/s13059-019-1841-x (PMC6833257; doi:10.1186/s13059-019-1841-x)
Supplement: Supplementary file 1 — Additional file 1 Figure S1. The results of experiments on limits of Mash Screen detection. [file 13059_2019_1841_MOESM1_ESM.pdf]

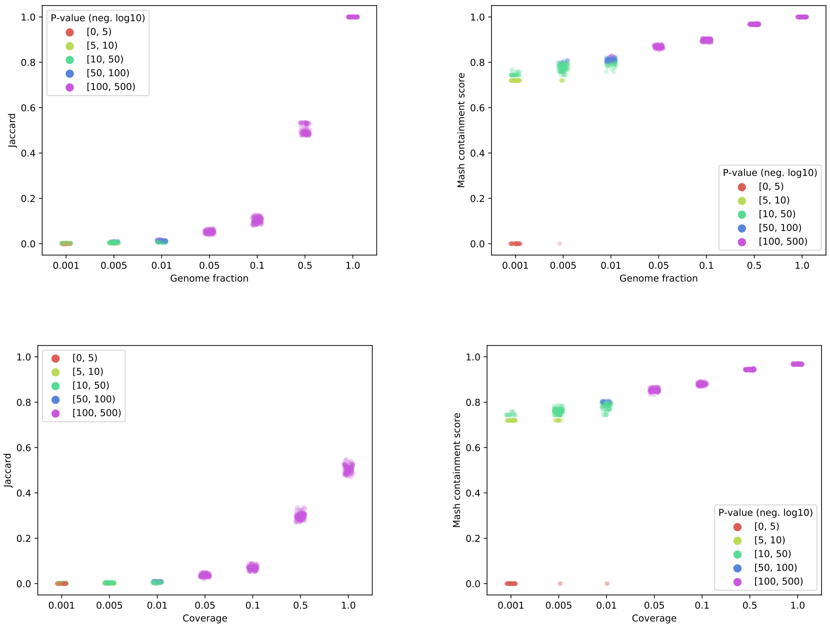

Figure S1 Relation of Mash containment scores to genome coverage. Experiments were performed by screening samples of an *E. coli* genome and screen against the complete reference genome. In the top charts ("Genome fraction" on x-axis), contiguous substrings of the genome were used, with lengths corresponding to the indicated fractions of the complete genome length. In the bottom charts ("Coverage" on x-axis), the indicated value was provided as the target coverage for simulating reads, which were then given to Mash Screen. The raw Jaccard scores (left plots) correspond closely to the actual fraction taken for contiguous subsequences, or to roughly half the read coverage (due to sequencing errors). The Mash containment score (right plots), however, is closer to the true identity than the true fraction contained because it models k-mer survival through mutational divergence rather than low coverage or structural change. Reads were simulated with ART (version "Mount Rainier"), using `-l 100` (100-base read length) and `-ss HS20` (Illumina HiSeq 2000 platform profile).
