# Supplementary material for: Mash Screen: high-throughput sequence containment estimation for genome discovery
Source: Genome Biol. 2019 Nov 5;20:232. doi: 10.1186/s13059-019-1841-x (PMC6833257; doi:10.1186/s13059-019-1841-x)
Supplement: Supplementary file 2 — Additional file 2 Review history. [file 13059_2019_1841_MOESM2_ESM.docx]

Review History

**First round of review**

**Reviewer 1**

**Were you able to assess all statistics in the manuscript, including the appropriateness of statistical tests used?** No.

**Were you able to directly test the methods?** Yes.

**Comments to author:**

The authors introduce "Mash Screen", a further development of their previously published algorithm "Mash". Mash is an extremely efficient algorithm to estimate the distance between two genome sequences. In the recent AFproject study (Zielezinski et al, bioRxiv 10.1101/611137), it was shown that Mash produces high-quality results.

A fundamental limitation of Mash is, however, that the program assumes that the compared sequences are homologous over their entire length. This is similar for many other alignment-free distance estimators. Mash Screen, by contrast, can estimate the level of "containment" of one genome in a set of reads (or metagenome).

The proposed approach is, to my knowledge, novel, the paper is well written and clear, and the results can be expected to be very useful in genome research.

Minor points:

- I think that the manuscript structure with "Methods" and "Results" that is common in the life sciences, is not suitable for a bioinfo or CS paper. But if the publisher insists on this structure, there is nothing that one can do.

- k (in k-mer) is a variable, so it should be in mathematical mode (italics).


**Reviewer 2**

**Were you able to assess all statistics in the manuscript, including the appropriateness of statistical tests used?** Yes: checked.

**Were you able to directly test the methods?** No.

**Comments to author:**

This study describes Mash Screen, a modification of the MinHash algorithm for alignment-free, k-mer-based genome comparison. While MinHash estimates the Jaccard index 
 j(A,B) = |A and B| / |A or B| 
as measure of resemblance of two genomes, Mash Screen estimates a genome containment score,
 c(A,B) = |A and B| / |A| . 
Here, A and B are set of k-mers occurring in (meta)genomes a and b, respectively. The tool is designed to compute the containment of all RefSeq genomes (the a's) in one or more metagenomes (the b's).

The authors searched with Mash Screen all 103,000 RefSeq genomes and plasmids through 145,000 metagenomes totaling 4 PB of memory, taking 0.5 million hours of CPU time. While the tool contains little algorithmic novelty and does not appear to show impressive speed in comparison to published tools, I consider this dataset to be of great practical importance for the metagenomics community and beyond.

Major points

1. Mash Screen is not compared with any competitor tool, and the reasons the paper give is this:
"These prior containment estimation methods have all sought to compress and/or index a large collection of sequencing reads, with the goal of enabling rapid sequence search across them (e.g. given a reference database of metagenomes, identify all those containing some query sequence). This is an important problem, but in this paper we consider the converse: given a reference database of genomes, identify all those contained in some query metagenome."

This problem should be addressable with other tools such as MMseqs (Steinegger et al, Nature Biotech 2017) or BIGSI (Bradley et al, Nature Biotech 2019). Both of these tools could index the RefSeq genomes as the database and search with the metagenomes through this database. MMseqs can search with the automatically six-frame translated reads through the RefSeq genomes database. It can also reformat the results to sort them by RefSeq Genomes and proteins instead of by translated metagenomic reads. In that way, the fraction of RefSeq sequences with a match in the metagenomic sample and their coverage by the reads can be found.

The speed of Mash Screen is surprisingly slow. From the numbers given, it processes 2 to 3 Mbp or reads per second, or around 1 bp per 100 clock cycles. (Why that slow?) From our experience with MMseqs, this tool is comparably fast in its "faster" mode (mmseqs prefilter --k-score 115) but probably is better at finding more diverged genomes. 

It would be important to see a quantitative comparison of speed and result quality with MMseqs and, if possible, BIGSI. 


2. Considering the potentially large usefulness of the results of the massive SRA search, a simple server should be set up to facilitate online searches of these results. 


3. Figure 4 is hard to understand. How can so many genes with Mash containment scores above 0.5 have such a low read coverage and such a low consensus identity in alignments with Diamonds? Doesn't it mean that the containment scores are dangerously optimistic? Might it be better to plot the logarithm of the Mash Screen P-value on the y-axis instead of the containment score?


Minor points

4. The definitions of Mash containment scores are contradictory. On page 3, line 23, it is C_k(A,B), whereas on page 10, line 28, it is given as c_k(a,b)^(1/k). Please also make the capitalization consistent throughout. Also, it is not explained unambiguously how the "Mash distance" is defined. Is it the Jaccard index j_k(a,b) or something else? If yes, why is it given a second name, D_k(A,A'), on page 3, line 23 ?

5. Missing numbers/information: 
* What sketch size is used by default and in your experiments. 
* What is the memory requirement of Mash Screen? How much memory was taken by the 103,000 RefSeq genomes? 
* Page 3, lines 36-38, page 6 lines 29-33: On how many cores, CPUs, and servers and on what type of CPUs was the time measured?
* In what language is Mash Screen implemented?
* What hash function is used?
* What map or hash library or implementation is used?

6. Figure 3: The Mash Distance on the x axis is not explained clearly in the caption.

7. Page 3, lines 32 and 33: "... those genomes sharing at least one sketch value with a known Shakya constituent were collected, resulting in 82,086 genomes." 
Why was that done? It would not be done in practice when applying Mash Screen, except if a utility tool is provided for that purpose.

8. Equation (4) is wrong. It refers to the case of drawing n but not necessarily distinct k-mers out of a pool of equiprobable k-mers. It becomes evident that the equation is wrong if one sets n = |Sigma|^k, in which case the result and not 1, as it should be, but instead 1 - exp(-1) = 0.63. 
The correct equation is simply
   r = n / |Sigma|^k.

9. Page 12, line 17: The correct equation is 
   n = 2^b (s/v),
not
   n = 2bs/v.

10. Missing words:
* Page 2, line 47: *to* transcriptomes
* Page 7, line 23: exposed *to*
* Page 8, line 44: It may *be*
* Page 9, line 58, form *of* locality
* Page 11, line 14: matches **at least** x out of 
* Page 12, line 57: screened *through* all known

**Reviewer 3**

**Were you able to assess all statistics in the manuscript, including the appropriateness of statistical tests used? Yes:** Much of the manuscript relies on statistical measures which have been defined previously and have been used appropriately here. Further, in my testing on data-sets not used within the manuscript, the introduced containment measure C_k(a,b) and significance threshold p(x; s; r) are valid and I found I could produce valid query results analogous to those found in the manuscript.

**Were you able to directly test the methods?** Yes.

**Comments to author:**

Are the methods appropriate to the aims of the study, are they well described, and are necessary controls included?

  Yes.

Are the conclusions adequately supported by the data shown?

  Yes.

Are sufficient details provided to allow replication and comparison with related analyses that may have been performed?

  Yes, all the required data and scripts are easily obtained and run.

Does the method perform better than existing methods (as demonstrated by direct comparison with available methods)?

  Yes. This new method, Mash Screen, solves the previously proposed containment query problem in a very effective manner. As the authors have stated, this method approaches an existing search problem in an inverse manner and so is not directly comparable.

Is the method likely to be of broad utility? Is any software component easy to install and use?

  Yes. This new method, Mash Screen, has wisely been added to an already mature and well-documented software project, which is the ideal. The whole project is easily cloned from its public archive and built from scratch. The project is also regularly released in a pre-built dependency-free form, making for easy non-expert installation.

Is the paper of broad interest to others in the field, or of outstanding interest to a broad audience of biologists?

  Yes. The rapidly growing size of public biological data archives makes highly space-efficient and fast search queries critically important in biological research. Containment queries, such as accomplished with Mash Screen, address a significant shortcoming of existing kmer sketch approaches which assess resemblance and whose value is the already well established. This was well explained in the manuscript.

Comments to authors

This is a well-written article, which effectively outlines the problem to be solved using language which should be tractable and impactful to a wide audience. The validation experiments are convincing and concise. The experiment which screened the whole SRA catalog against RefSeq very successfully demonstrated the method's utility. The final study on the distribution of human polyomaviruses was a great reminder of the errors which may exist within our reference databases and how logical thinking and effective big-data methods can uncover them. The 6-frame amino acid containment query is an interesting use-case, leaving users to perhaps think carefully about how they should construct a meaningful reference proteome.

1. Though the authors have stated that the method approaches the containment problem in a converse fashion to preexisting tools, any type of practical comparison between these other tools and Mash Screen would be of interest. This could simply be, for equivalent -- albeit inverse -- queries, the required disk space of the resulting indexes and runtime memory. 

2. When building from source, the autoconf based compilation was straight-forward, but it would be handy if users had control of the choice to statically or dynamically link to the CapnProto libraries (libcapnp, libkj). Such control is available when linking to the GNU Scientific Library (libgsl, libgslcblas) using the "--with-static-gsl" option. I mention this because not all Linux distributions provide static versions of CapnProto and as a result, users with these distributions must either modify the makefile or separately install a static version of CapnProto.

3. Figure 4: The three situations where points fall along x=0 or 1, or y=0 do not seem to have been discussed.

Minor notes

4. Page 1, line 43: The use of a tilde to represent "approximately" (~21) does not typeset well and might be better as simply "approx. 21" or an alternative symbol such as Unicode 2248 (≈) (latex \approx)

5. Page 1, line 59: Perhaps the reference cited later for resemblance and containment (8) could first be cited here.

6. Page 2, line 47: insert "to" between "applicable transcriptomes"

7. Page 2, line 48: it would be helpful to cite articles for these stated applications.

8. Page 8, line 29-34: In the opening paragraph of the discussion, the important point being made by the authors -- not to mistake Mash Screen as a tool for composition analysis -- could be clearer. I take it that they are referring to situations of a sketch database contains similar many strains.

9. Page 9, line 9: Citing an example of such tools, which "rely on mapping metagenomic sequence reads to reference genome databases" would be of value to readers.

10. Page 9, line 42: Is the term "the mixture" synonymous with the term "pool" used in the software? Would a single term be preferable?

11. Page 9, line 56: Perhaps "agreement" rather than "compatibility"?

12. Page 9, line 57: Replace "improves" with "improving".

13. Page 9: line 58: "This is because" rather than "Because" would maintain more connection between this sentence and the last.

14. Page 12, line 11: Perhaps "negating" rather than "erasing"?

15. Page 12, line 57: Missing word (against) "further screened against all known viruses".

16. Page 14, line 13: The reference (Koslicki et al, 2017) should be updated to reflect the article's recent publication (doi:10.1016/j.amc.2019.02.018)

**Reviewer 4**

**Were you able to assess all statistics in the manuscript, including the appropriateness of statistical tests used? Yes:** A p-value computation is given for the containment score under the null hypothesis that the sequences are unrelated. It would be good to adjust this p-value to reflect sequence composition, as it currently assumes equal base frequencies in estimating the chance of a random k-mer match; however, to first order, the computation seems appropriate.

**Were you able to directly test the methods?** No.

**Comments to author:**

This paper describes Mash Screen, an extension of the Mash software for sketch-based estimation of sequence similarity to measure containment of one sequence in another.  Rather than compare sketches, the screening approach measures the fraction of k-mers in one sketch that is present in an entire genome or metagenome.  This approach allows much more accurate estimation of shared k-mer count when one sequence (the one being sketched) is much shorter than the other.  The authors apply their approach to screen every metagenome in the NIH Sequence Read Archive for the presence of every genome and plasmid in RefSeq, and to detect polyoma viruses in human metagenomic samples.

This is a valuable extension of sketch-based global similarity measurement to more localized detection.  The primary benefit is quantitative detection of genomes or fragments thereof in large metagenomic data sets much faster than by traditional alignment or exhaustive k-mer counting, thanks to the sampling nature of sketching. The main downside is that only one side of the comparison can be sketched; the other (larger) side must be streamed or hashed in some way, requiring linear work to process. This cost may be acceptable for occasional use, as with the authors' proposed periodic all-vs-all comparison of SRA vs RefSeq, or with a large cluster of machines that can subdivide the sequences being streamed.  If, however, one were to support an application in which users could sketch arbitrary large queries and ask about their containment, efficiency might demand preprocessing of the metagenomes using, e.g., one of the Bloom filter-based approaches cited by the authors.

Validation mainly involves some very large-scale comparisons which would be computationally much more difficult, if not infeasible, without sketching.  However, these comparisons are mostly limited to detecting highly contained sequences.  It's hard to tell if or how well the approach would work at detecting containment of relatively weak homologues to a given query.  I'd like to see some additional experiments, and possibly adjustments to the p-value calculation (suggested below), that address this use case.

The exposition was fairly clear overall, with a few areas that would benefit from definition of terms referenced from other work.  

Specific comments follow:

* pg 2, line 47 -- should read "applicable *to* transcriptomes"

* pg 3, top of page -- I don't understand the phrase "that which is contained within the metagenome".

* pg 3, line 23 -- the Mash distance D is mentioned but not defined, nor does it appear to be defined in the methods.  While it was defined in another paper, the definition should be restated here, given that it is used extensively in the experiments of Section 2.

* pg 3, line 37 -- if you are going to quote a performance number here, please also state the k-mer size and sketch size used in the same place.

* Section 2.2: how do you tell the difference between contamination and legitimate horizontal transfer or other homology?  the latter would presumably not entail finding the majority of a second genome in the read set, as in this case, but what is the lower bound on the tool's ability to reliably detect a small (but possibly important) part of one organism's genome in the larger sample?   For example, could one detect that a metagenome does not contain most of a particular organism but does contain a known pathogenicity island that is present in that organism's genome?  Some experiments to illustrate the lower limit of significance or detection for partial matches to a genome in a metagenome (essentially the inverse of Table 3, which looks at increasingly higher containment scores) would be helpful.  It would also increase confidence in the sensitivity of the screen for viruses shown in Table 5.

*  Sectiion 2.4: would it be worthwhile to rapidly eliminate from consideration those metagenomes that have a very low containment score for a given sketch? For example, one could precompute a Bloom filter per metagenome.  Such precomputation could be useful if not all sketches can be processed in one pass (did you really count the k-mers from the sketches for every sequence in RefSeq in a single pass?), or if the screen might be done with additional sketches in future.

* pg 7, line 23 -- should  be "exposed *to* human polyomaviruses..."

* pg 7, line 36 -- define k-mer multiplicity

* Section 3.1 -- what is the lower limit of sequence size below which sketching provides no practical speed benefit over using all k-mers?  Presumably it is not just the size of a sketch, as there is some overhead to sketching, and the marginal cost to process a couple more k-mers in the streaming phase is small.

* Section 4.1.2 -- the significance scores in (4)/(5) could be modified to account for the base frequencies of the sequences being compared. Similar adjustments are used in, e.g., BLAST.  Although the tests done here mainly detect extremely highly contained (and therefore highly significant) sequences, it might be worth applying what seems like a simple adjustment.  The suggested experiments above regarding the lower limit of detection could benefit from such an improvement.

On a related issue, would it be possible to estimate detection power as a function of divergence between the query sequence and the actual sequence contained in the metagenome?  Previous work on comparing long k-mer spectra (without sketching) to estimate similarity used a Poisson model of mutation to estimate the likely fraction of k-mers shared between two sequences given a certain rate of divergence.  The same model could be applied here.
 
* Section 4.2.1 -- can you quantify the degree of compression (in terms of # of distinct k-mers) obtained on your SRA vs RefSeq experiment?  That number would back up the claimed  benefit of sketching in this section.

**Authors Response**

**Point-by-point responses to the reviewers’ comments:**

*>We thank all four reviewers for their thoughtful and thorough recommendations. We have addressed the concerns, to the best of our ability, in comments below and in the manuscript.*

**Reviewer #1:**

The authors introduce "Mash Screen", a further development of their previously published algorithm "Mash". Mash is an extremely efficient algorithm to estimate the distance between two genome sequences. In the recent AFproject study (Zielezinski et al, bioRxiv 10.1101/611137), it was shown that Mash produces highquality results. A fundamental limitation of Mash is, however, that the program assumes that the compared sequences are homologous over their entire length. This is similar for many other alignment-free distance estimators. Mash Screen, by contrast, can estimate the level of "containment" of one genome in a set of reads (or metagenome). The proposed approach is, to my knowledge, novel, the paper is well written and clear, and the results can be expected to be very useful in genome research.

*>Thank you for your positive comments.*

Minor points: - I think that the manuscript structure with "Methods" and "Results" that is common in the life sciences, is not suitable for a bioinfo or CS paper. But if the publisher insists on this structure, there is nothing that one can do.

*>We agree there is some awkwardness in fitting a computational method to this format, but we ultimately felt it was best to conform to the journal’s guidelines.*

- k (in k-mer) is a variable, so it should be in mathematical mode (italics).

*>Italics have been applied to k-mer throughout the text.*

**Reviewer #2:**

This study describes Mash Screen, a modification of the MinHash algorithm for alignment-free, k-mer-based genome comparison. While MinHash estimates the Jaccard index

j(A,B) = |A and B| / |A or B|

as measure of resemblance of two genomes, Mash Screen estimates a genome containment score,

c(A,B) = |A and B| / |A| .

Here, A and B are set of k-mers occurring in (meta)genomes a and b, respectively. The tool is designed to compute the containment of all RefSeq genomes (the a's) in one or more metagenomes (the b's).

The authors searched with Mash Screen all 103,000 RefSeq genomes and plasmids through 145,000 metagenomes totaling 4 PB of memory, taking 0.5 million hours of CPU time. While the tool contains little algorithmic novelty and does not appear to show impressive speed in comparison to published tools, I consider this dataset to be of great practical importance for the metagenomics community and beyond.

*>We note that the 4PB listed in the text is the size of raw files, which do not need to be kept in memory. We have clarified this in the text, including added memory requirements to the appropriate sections, as requested below.*

Major points 1. Mash Screen is not compared with any competitor tool, and the reasons the paper give is this:

"These prior containment estimation methods have all sought to compress and/or index a large collection of sequencing reads, with the goal of enabling rapid sequence search across them (e.g. given a reference database of metagenomes, identify all those containing some query sequence). This is an important problem, but in this paper we consider the converse: given a reference database of genomes, identify all those contained in some query metagenome."

This problem should be addressable with other tools such as MMseqs (Steinegger et al, Nature Biotech 2017) or BIGSI (Bradley et al, Nature Biotech 2019). Both of these tools could index the RefSeq genomes as the database and search with the metagenomes through this database. MMseqs can search with the automatically sixframe translated reads through the RefSeq genomes database. It can also reformat the results to sort them by RefSeq Genomes and proteins instead of by translated metagenomic reads. In that way, the fraction of RefSeq sequences with a match in the metagenomic sample and their coverage by the reads can be found.

*>We agree that MMSeqs, or, more generally, any alignment tool, could theoretically be used to solve the containment problem as suggested, and likely with higher sensitivity. In practice, however, for large databases, this requires computing resources that are not widely available and could not scale to the type of applications that we demonstrate. As previously noted in the manuscript, Mash Screen sacrifices some sensitivity for speed, enabling the analysis of very large datasets. As MMSeqs was neither intended for the same purpose as Mash Screen nor, in our tests, practically repurposable for it, we did not think it was appropriate to compare against in the text. As detailed below, these comparisons would be unfairly harsh on MMSeqs.*

The speed of Mash Screen is surprisingly slow. From the numbers given, it processes 2 to 3 Mbp or reads per second, or around 1 bp per 100 clock cycles. (Why that slow?) From our experience with MMseqs, this tool is comparably fast in its "faster" mode (mmseqs prefilter --k-score 115) but probably is better at finding more diverged genomes.

*>We agree there is substantial room for optimizing our implementation (e.g. via better hash table implementations mentioned in the discussion) that will further improve efficiency in future releases.*

It would be important to see a quantitative comparison of speed and result quality with MMseqs and, if possible, BIGSI.

*>We attempted to run MMSeqs on the 11 gigabase Shakya et al. Illumina read set used in the experiment described in section 2.1. Mash Screen processed this run in 61 CPU-minutes using a maximum of 11 GB of RAM, operating on a 1.2 GB RefSeq sketch with a total output of 25 MB.*

*As suggested, we created a database of translated reads and a database of proteins from the RefSeq genomes.The RefSeq index took 232 GB of disk (181 GB using -- compressed).*

*We ran MMSeqs prefilter on the same server as for Mash Screen (dual Intel Xeon E5- 2698 v4 2.20 GHz CPU, 40 cores, 80 hyperthreads with 1 TB RAM and 3.6 TB of scratch space), and with the same number of threads (32), using local disk for scratch and with the recommended sensitivity settings:*

*“mmseqs prefilter --threads 32 -s 1.0 --k-score 115 --local-tmp /scratch/tmp/ reads.trans refseq.db prefilter”*

*While running, the tool used more than 500 GB of RAM, and more than 3 TB of temporary files, producing this warning:*

*“Hard disk might not have enough free space (3614437511168 bytes left).The prefilter result might need maximal 3794597616600 bytes.”*

*After running for a week (wall-clock), with close to full utilization of its 32 cpus, the progress bar indicated it would take more than another week to complete (prefiltering alone, with no alignment). Some of our attempts crashed after, presumably, running out of disk space, even with terabytes of scratch provided. We also attempted various configurations, such as different values for --k-score, using the ‘search’ workflow instead of prefiltering separately, and swapping the reference and target. Ultimately, we were not able to get the tool to produce results in a reasonable timeframe for these revisions.*

*It was also not possible to compare against BIGSI, because it is solving a different problem. Like Mash Screen, BIGSI detects whether a shorter query is contained in any of its indexed sequences. However, BIGSI can only index isolate genomes, not metagenomes, and so cannot operate on the types of data presented in our manuscript. Though the authors of BIGSI mention indexing of metagenomic samples for future work, BIGSI currently requires a fixed, estimated genome length for its* *indices, limiting it to containment of shorter sequences within microbial isolates. E.g. from the BIGSI paper: “A limitation of BIGSI is that Kmax, the maximum number of kmers per dataset, must be set in advance.”*

2. Considering the potentially large usefulness of the results of the massive SRA search, a simple server should be set up to facilitate online searches of these results.

*>This is a great idea and something we plan to do. However, due to the processes we must go through as a government entity in order to set up a public-facing website, we were not able to get this live in the timeframe of these revisions. In the interim, we have provided a script and associated README to make queries on the raw data easier. We are also in discussions with NCBI to link similar results directly from the SRA webpages.*

3. Figure 4 is hard to understand. How can so many genes with Mash containment scores above 0.5 have such a low read coverage and such a low consensus identity in alignments with Diamonds? Doesn't it mean that the containment scores are dangerously optimistic? Might it be better to plot the logarithm of the Mash Screen Pvalue on the y-axis instead of the containment score?

*>As suggested, we have incorporated the Mash Screen p-value into this plot (though as color mapping rather than the y-axis to preserve the direct comparison). In the new plot it can be seen that the overestimated scores on the left of the plot have relatively high p-values and could be filtered out with a reasonable threshold.*

*>We also investigated the points in the extreme upper left corner and found that they were caused by real mappings that had been missed by DIAMOND. Increasing the sensitivity settings of DIAMOND moved most of these closer to the diagonal, and the few that remain represent legitimate short or low complexity hits that DIAMOND missed due to its own internal heuristics.*

Minor points 4. The definitions of Mash containment scores are contradictory. On page 3, line 23, it is C_k(A,B), whereas on page 10, line 28, it is given as c_k(a,b)^(1/k). Please also make the capitalization consistent throughout. Also, it is not explained unambiguously how the "Mash distance" is defined. Is it the Jaccard index j_k(a,b) or something else? If yes, why is it given a second name, D_k(A,A'), on page 3, line 23 ?

*>The Mash scores (Mash distance or Mash containment score) are derived from raw set indices (Jaccard or its counterpart “containment index” as defined by Broder) by modeling k-mer survival to estimate a mutation rate. We agree that the use of capitalization to distinguish the Mash containment score (capital C) from the set containment index (lowercase c) was confusing, and have switched to using MC (containment) and MD (distance) for the Mash scores. To clarify these definitions, the Mash distance as defined in Ondov et al. 2015 is now also restated in 4.1.*

5. Missing numbers/information: * What sketch size is used by default and in your experiments.

*>A sketch size of 1,000 was used throughout for the Mash Screen results. This is now noted in the text where appropriate.*

* What is the memory requirement of Mash Screen? How much memory was taken by the 103,000 RefSeq genomes?

*>Mash Screen used a peak of 11 GB searching against RefSeq for the Shakya metagenome, most of which was taken by the RefSeq index. This is now mentioned in section 2.1. The typical usage for SRA runs (10-15GB) is noted in 2.4.*

* Page 3, lines 36-38, page 6 lines 29-33: On how many cores, CPUs, and servers and on what type of CPUs was the time measured?

*>This now refers to section 4.3.1, where the details are provided.*

* In what language is Mash Screen implemented?

*>C++. Now noted in section 4.2.*

* What hash function is used?

*>To maintain consistency with the prior versions of Mash, MurmurHash3 was used. This is now noted and cited in section 4.2:*

*>“For compatibility with existing Mash sketches, MurmurHash3 is used to hash k-mers, as in Ondov et al.”*

* What map or hash library or implementation is used?

*>STL unordered_map. Now noted in section 4.2.1.*

6. Figure 3: The Mash Distance on the x axis is not explained clearly in the caption.

*>The caption has been updated to more directly describe the x-axis:*

*>“The x-axis is the Mash Distance to the nearest genome in the Shakya synthetic metagenome, which serves as an expected containment score.”*

7. Page 3, lines 32 and 33: "... those genomes sharing at least one sketch value with a known Shakya constituent were collected, resulting in 82,086 genomes." Why was that done? It would not be done in practice when applying Mash Screen, except if a utility tool is provided for that purpose.

*>This was done because the Mash distance is not a perfect benchmark, given its limited sensitivity. For example, one would expect a phage contained in a bacterial genome to be picked up by Mash Screen, but not by the Mash Distance, which is sensitive to size differences between the sequences being compared. Such phage genomes could appear, spuriously, as false positives in this analysis.*

*>We agree, however, that simply cutting off the reference scores (x-axis) at their limit of detection was misleading and could have hidden real false positives. Thus, we have updated this analysis by restricting the genomes we consider by their size, rather than whether Mash detected them. This measure, combined with increasing the sketch size used for Mash Distance to 100,000, allowed us to show more definitively that false positives do not appear at more diverged distances (with the exception, of course, of contaminants). While these measures would not be taken when running Mash Screen normally, we note that this analysis is not intended to show Mash Screen’s limits of detection, but rather its correlation with Mash distances. Thus, some filtering was necessary to obtain a reasonable set of expected values.*

8. Equation (4) is wrong. It refers to the case of drawing n but not necessarily distinct kmers out of a pool of equiprobable k-mers. It becomes evident that the equation is wrong if one sets n = |Sigma|^k, in which case the result and not 1, as it should be, but instead 1 - exp(-1) = 0.63.

The correct equation is simply r = n / |Sigma|^k.

*>Thank you for catching this subtle error. Equation 4 was originally defined with the idea that a metagenome is a random stream of k-mers that we were sampling with replacement. However, the reviewer is correct that we are currently estimating the number of distinct k-mers in the metagenome, rather than the total number of k-mers. We think using the distinct k-mer estimate is the most proper for computing the pvalue. Therefore, we agree that it makes more sense to use the reviewer’s suggested formulation for eq 4 that reflects sampling without replacement. We have updated the p-value formula both in the text and in the latest version of the code. In most cases, the two formulas yield similar results.*

*>The only figure in the paper that displays p-values, Figure 4, has been updated to use this new formula. Rerunning the SRA experiment was not feasible in the time frame of these revisions, but we plan to release new versions of the supplemental SRA tables filtered on this new p-value when they are available. While Figure 5 is based on these tables and thus still uses the old p-value for filtering, we ran tests and found that pvalues near the cut-off used (1e-100) were nearly identical between the old and new formulas, and so this correction does not affect any of the conclusions presented in the paper. The older version of Mash Screen used for this experiment has been noted in the text for reproducibility.*

9. Page 12, line 17: The correct equation is n = 2^b (s/v), not n = 2bs/v.

*>Thank you for catching this typesetting error. We have corrected it.*

10. Missing words:

* Page 2, line 47: *to* transcriptomes

* Page 7, line 23: exposed *to*

* Page 8, line 44: It may *be*

* Page 9, line 58, form *of* locality

* Page 11, line 14: matches **at least** x out of

* Page 12, line 57: screened *through* all known

*>These errors have been corrected.*

**Reviewer #3:**

Are the methods appropriate to the aims of the study, are they well described, and are necessary controls included? Yes.

Are the conclusions adequately supported by the data shown? Yes.

Are sufficient details provided to allow replication and comparison with related analyses that may have been performed? Yes, all the required data and scripts are easily obtained and run.

Does the method perform better than existing methods (as demonstrated by direct comparison with available methods)? Yes. This new method, Mash Screen, solves the previously proposed containment query problem in a very effective manner. As the authors have stated, this method approaches an existing search problem in an inverse manner and so is not directly comparable.

Is the method likely to be of broad utility? Is any software component easy to install and use? Yes. This new method, Mash Screen, has wisely been added to an already mature and well-documented software project, which is the ideal. The whole project is easily cloned from its public archive and built from scratch. The project is also regularly released in a pre-built dependency-free form, making for easy non-expert installation.

Is the paper of broad interest to others in the field, or of outstanding interest to a broad audience of biologists? Yes. The rapidly growing size of public biological data archives makes highly space efficient and fast search queries critically important in biological research. Containment queries, such as accomplished with Mash Screen, address a significant shortcoming of existing kmer sketch approaches which assess resemblance and whose value is the already well established. This was well explained in the manuscript.

Comments to authors

This is a well-written article, which effectively outlines the problem to be solved using language which should be tractable and impactful to a wide audience. The validation experiments are convincing and concise. The experiment which screened the whole SRA catalog against RefSeq very successfully demonstrated the method's utility. The final study on the distribution of human polyomaviruses was a great reminder of the errors which may exist within our reference databases and how logical thinking and effective big-data methods can uncover them. The 6-frame amino acid containment query is an interesting use-case, leaving users to perhaps think carefully about how they should construct a meaningful reference proteome.

1. Though the authors have stated that the method approaches the containment problem in a converse fashion to preexisting tools, any type of practical comparison between these other tools and Mash Screen would be of interest. This could simply be, for equivalent -- albeit inverse -- queries, the required disk space of the resulting indexes and runtime memory.

*>We have added text to this effect following Mash Screen’s performance in 2.1:*

*>“Though not directly comparable to the indexing methods mentioned above, which have both different approaches and applications, the database compression of Mash (950x in this case) is of a similar scale to that reported by Mantis (250x) or BIGSI (113x). Memory requirements of these tools tend to closely track the size of these indexes, and this is true of Mash Screen as well.”*

2. When building from source, the autoconf based compilation was straight-forward, but it would be handy if users had control of the choice to statically or dynamically link to the CapnProto libraries (libcapnp, libkj). Such control is available when linking to the GNU Scientific Library (libgsl, libgslcblas) using the "--with-static-gsl" option. I mention this because not all Linux distributions provide static versions of CapnProto and as a result, users with these distributions must either modify the makefile or separately install a static version of CapnProto.

*>For general users, Mash is intended to be downloaded directly as a binary, rather than built from source. We do, however, appreciate any feedback on the build process and plan to add this option in the future.*

3. Figure 4: The three situations where points fall along x=0 or 1, or y=0 do not seem to have been discussed.

*>The points in the upper left corner were due to real mappings that DIAMOND had missed in its default mode. Most of them move toward the main cluster with the sensitivity increased, and this has been updated in the figure. The points along the bottom are alignments that Mash Screen missed due to even spacing of mismatches, which knocked out all matching k-mers. This known sensitivity limitation is now discussed in the caption:*

*>“Points along the x-axis represent the limits of Mash Screen's sensitivity. For these genes, mismatches were common enough, and evenly distributed enough, to change all k-mers indexed by Mash Screen, which in this case were 9 amino acids long.”*

Minor notes

4. Page 1, line 43: The use of a tilde to represent "approximately" (~21) does not typeset well and might be better as simply "approx. 21" or an alternative symbol such as Unicode 2248 (≈) (latex \approx)

*>We have removed the tildes and used both suggested options depending on context.*

5. Page 1, line 59: Perhaps the reference cited later for resemblance and containment (8) could first be cited here.

6. Page 2, line 47: insert "to" between "applicable transcriptomes"

*>Corrected.*

7. Page 2, line 48: it would be helpful to cite articles for these stated applications.

*>We have reworked this section to pair the cited tools with their applications:*

*“For the specific application of searching transcriptomes, Bloom trees and Counting Quotient Filters have been proposed. Additionally, Bloom filter matrices have been used for searching within unassembled genome isolates.”*

8. Page 8, line 29-34: In the opening paragraph of the discussion, the important point being made by the authors -- not to mistake Mash Screen as a tool for composition analysis -- could be clearer. I take it that they are referring to situations of a sketch database contains similar many strains.

*>This is correct, and we have clarified using this example:*

*“For example, many different strains of a given species would be expected to have high containment scores even if only one strain is present in the mixture.”*

9. Page 9, line 9: Citing an example of such tools, which "rely on mapping metagenomic sequence reads to reference genome databases" would be of value to readers.

*>We have added several citations.*

10. Page 9, line 42: Is the term "the mixture" synonymous with the term "pool" used in the software? Would a single term be preferable?

*>We have changed “pool” to “mixture” in the software for consistency.*

11. Page 9, line 56: Perhaps "agreement" rather than "compatibility"?

*>We prefer “compatibility” since this is more to due with software interoperability than consistency of results.*

12. Page 9, line 57: Replace "improves" with "improving".

*>Corrected.*

13. Page 9: line 58: "This is because" rather than "Because" would maintain more connection between this sentence and the last.

*>This change would alter the meaning from what is intended.*

14. Page 12, line 11: Perhaps "negating" rather than "erasing"?

*>Agreed, changed.*

15. Page 12, line 57: Missing word (against) "further screened against all known viruses".

*>Added.*

16. Page 14, line 13: The reference (Koslicki et al, 2017) should be updated to reflect the article's recent publication (doi:10.1016/j.amc.2019.02.018)

*>The citation has been updated.*

**Reviewer #4:** This paper describes Mash Screen, an extension of the Mash software for sketch-based estimation of sequence similarity to measure containment of one sequence in another. Rather than compare sketches, the screening approach measures the fraction of k-mers in one sketch that is present in an entire genome or metagenome. This approach allows much more accurate estimation of shared k-mer count when one sequence (the one being sketched) is much shorter than the other. The authors apply their approach to screen every metagenome in the NIH Sequence Read Archive for the presence of every genome and plasmid in RefSeq, and to detect polyoma viruses in human metagenomic samples.

This is a valuable extension of sketch-based global similarity measurement to more localized detection. The primary benefit is quantitative detection of genomes or fragments thereof in large metagenomic data sets much faster than by traditional alignment or exhaustive k-mer counting, thanks to the sampling nature of sketching. The main downside is that only one side of the comparison can be sketched; the other (larger) side must be streamed or hashed in some way, requiring linear work to process. This cost may be acceptable for occasional use, as with the authors' proposed periodic all-vs-all comparison of SRA vs RefSeq, or with a large cluster of machines that can subdivide the sequences being streamed. If, however, one were to support an application in which users could sketch arbitrary large queries and ask about their containment, efficiency might demand preprocessing of the metagenomes using, e.g., one of the Bloom filter-based approaches cited by the authors.

Validation mainly involves some very large-scale comparisons which would be computationally much more difficult, if not infeasible, without sketching. However, these comparisons are mostly limited to detecting highly contained sequences. It's hard to tell if or how well the approach would work at detecting containment of relatively weak homologues to a given query. I'd like to see some additional experiments, and possibly adjustments to the p-value calculation (suggested below), that address this use case.

The exposition was fairly clear overall, with a few areas that would benefit from definition of terms referenced from other work.

Specific comments follow:

* pg 2, line 47 -- should read "applicable *to* transcriptomes"

*>Fixed.*

* pg 3, top of page -- I don't understand the phrase "that which is contained within the metagenome".

*>Changed to “a sequence contained within the metagenome”.*

* pg 3, line 23 -- the Mash distance D is mentioned but not defined, nor does it appear to be defined in the methods. While it was defined in another paper, the definition should be restated here, given that it is used extensively in the experiments of Section 2.

*>The Mash distance is now explained in section 4.1.*

* pg 3, line 37 -- if you are going to quote a performance number here, please also state the k-mer size and sketch size used in the same place.

*>Defaults were used (k=2, s=1000); this has been noted.*

* Section 2.2: how do you tell the difference between contamination and legitimate horizontal transfer or other homology? the latter would presumably not entail finding the majority of a second genome in the read set, as in this case, but what is the lower bound on the tool's ability to reliably detect a small (but possibly important) part of one organism's genome in the larger sample? For example, could one detect that a metagenome does not contain most of a particular organism but does contain a known pathogenicity island that is present in that organism's genome? Some experiments to illustrate the lower limit of significance or detection for partial matches to a genome in a metagenome (essentially the inverse of Table 3, which looks at increasingly higher containment scores) would be helpful. It would also increase confidence in the sensitivity of the screen for viruses shown in Table 5.

*>Thank you for raising this important point. Indeed, high-identity local homologies (such as horizontally acquired elements) can be difficult to distinguish from low-identity genome-wide similarity on the basis of containment scores alone. In a real-world scenario we would typically validate weak containment scores by mapping reads to the relevant reference and checking if mapping coverage was uniform or restricted to only a few regions. We have added a discussion of this to the text:*

*>(Section 3.1) “Similarly, short, high-identity regions of a genome, such as horizontally transferred elements, are not distinguished from weak, overall similarity. The downstream analyses we have performed in our experiments, such as mapping reads to discovered genomes, thus remain a crucial part of deploying Mash Screen in practice.*

*>(Section 4.1) “Note that, like the Mash Distance, this formula does not attempt to separate divergence from missing sequence. In cases where the mixture contains small pieces of a genome, but at high identity (e.g. potentially with horizontal transfer), we expect the Mash containment score to be closer to identity than coverage (Supp. Fig. S1).”*

*>We have also added a supplemental figure showing the results of new experiments testing the lower limits of Mash Screen. Briefly, Mash Screen does pick up fairly small regions (or low coverage), but tends to report a score closer to the identity than the fraction of the genome.*

* Section 2.4: would it be worthwhile to rapidly eliminate from consideration those metagenomes that have a very low containment score for a given sketch? For example, one could precompute a Bloom filter per metagenome. Such precomputation could be useful if not all sketches can be processed in one pass (did you really count the k-mers from the sketches for every sequence in RefSeq in a single pass?), or if the screen might be done with additional sketches in future.

*>This indeed can be performed in a single pass, due both to the use of bottom sketching and the overlap in hashes among individual RefSeq sketches (related to comments about section 4.2.1 below). The bloom filter idea, however, could be useful for making incremental updates of the SRA vs. RefSeq table more efficient, and we appreciate the suggestion.*

* pg 7, line 23 -- should be "exposed *to* human polyomaviruses..."

*>Corrected.*

* pg 7, line 36 -- define k-mer multiplicity

*>Since we also mention this earlier in section 2.4, we now define it there and reference in this line.*

* Section 3.1 -- what is the lower limit of sequence size below which sketching provides no practical speed benefit over using all k-mers? Presumably it is not just the size of a sketch, as there is some overhead to sketching, and the marginal cost to process a couple more k-mers in the streaming phase is small.

*>It is difficult to assess the efficiency inflection exactly, since a k-mer based tool would have different architecture. However, we have expanded the discussion in 3.1:*

*>“In these cases, the sketch of a gene can be as large as the gene itself. As a sketch, by default, uses 8 bytes for each hash, a sequence would have to be roughly an order of magnitude larger in length than the sketch size (1000 by default) to accrue indexing storage benefits. Further, hashing k-mers in the input stream is a nontrivial component of the computational cost, and this step, which is important for probabilistic estimation, would not be necessary when simply counting all k-mers.”*

* Section 4.1.2 -- the significance scores in (4)/(5) could be modified to account for the base frequencies of the sequences being compared. Similar adjustments are used in, e.g., BLAST. Although the tests done here mainly detect extremely highly contained (and therefore highly significant) sequences, it might be worth applying what seems like a simple adjustment. The suggested experiments above regarding the lower limit of detection could benefit from such an improvement.

*>We appreciate the suggestion and have also considered this. However, as noted, the typical use case for Mash Screen is to detect highly significant hits rather than distant relationships. For this purpose, the current p-value serves to alert users of clearly insignificant results that arise when screening large metagenomes using small k-mer sizes, and as the revised Figure 4 shows, it does a decent job of stratifying the matches. We leave a more sophisticated p-value as future work if a new use case is identified that requires it.*

On a related issue, would it be possible to estimate detection power as a function of divergence between the query sequence and the actual sequence contained in the metagenome? Previous work on comparing long k-mer spectra (without sketching) to estimate similarity used a Poisson model of mutation to estimate the likely fraction of kmers shared between two sequences given a certain rate of divergence. The same model could be applied here.

*>An estimation of this kind was previously available for Mash distance estimates using the ‘bounds’ command, and we have now updated this command to show values for screen scores as well as distances. For converting the Jaccard index to a mutational distance, the Mash distance function uses a Poisson model, similar to those noted by the reviewer. For Mash Screen, we adopted a related binomial model, and found the two to be largely interchangeable. The ‘bounds’ command is now available for both. This has also been noted in the manuscript in section 4.1:*

*>“Error bounds on this estimate for various values of s and k can be computed using the bounds subcommand.”*

* Section 4.2.1 -- can you quantify the degree of compression (in terms of # of distinct k-mers) obtained on your SRA vs RefSeq experiment? That number would back up the claimed benefits of sketching in this section.

*>The compression, roughly an order of magnitude, has been added to the end of this section.*

* A p-value computation is given for the containment score under the null hypothesis that the sequences are unrelated. It would be good to adjust this p-value to reflect sequence composition, as it currently assumes equal base frequencies in estimating the chance of a random k-mer match; however, to first order, the computation seems appropriate.

*>See our response to the related comment above. We have not yet encountered a use case that requires a more sophisticated p-value and the current formulation has been sufficient. We agree that considering sequence composition would be the first step to improving it, but leave this as future work.*

**Second round of review**

**Reviewer 2**

Nice work.

**Reviewer 4**

The authors have corrected a number of small errors and addressed issues raised about the underlying method and its exposition.  The main potential issues at this time are (1) the SRA analysis was done with an older version of the code, but the newer version corrects a statistical error identified by a reviewer, and (2) the authors have not added comparisons to other tools to their paper.  With respect to the first issue, the only impact would likely be on matches with marginal p-values, which does not change the overall conclusion that it is feasible to do the analysis; I am happy with the authors' intent to produce a revised data set for future use.  With respect to the second issue, the authors tried to use a competing tool but found that it used an infeasible amount of storage and time (the latter probably because of needing to access the index on secondary storage).  I will defer to the reviewer who raised the concern, but it does seem like they made a reasonable attempt to address it, and that the competing tool cannot be used off the shelf for their analysis.

Overall, the authors have done a good job of responding to comments.
